# Attosecond-resolution Hong-Ou-Mandel interferometry

**DOI:** 10.1126/sciadv.aap9416

**Published:** 2018-05-04

**Authors:** Ashley Lyons, George C. Knee, Eliot Bolduc, Thomas Roger, Jonathan Leach, Erik M. Gauger, Daniele Faccio

**Affiliations:** 1 School of Engineering and Physical Sciences, Heriot-Watt University, Edinburgh EH14 4AS, UK.; 2 School of Physics and Astronomy, University of Glasgow, Glasgow G12 8QQ, UK.; 3 Department of Physics, University of Warwick, Coventry CV4 7AL, UK.

## Abstract

When two indistinguishable photons are each incident on separate input ports of a beamsplitter, they “bunch” deterministically, exiting via the same port as a direct consequence of their bosonic nature. This two-photon interference effect has long-held the potential for application in precision measurement of time delays, such as those induced by transparent specimens with unknown thickness profiles. However, the technique has never achieved resolutions significantly better than the few-femtosecond (micrometer) scale other than in a common-path geometry that severely limits applications. We develop the precision of Hong-Ou-Mandel interferometry toward the ultimate limits dictated by statistical estimation theory, achieving few-attosecond (or nanometer path length) scale resolutions in a dual-arm geometry, thus providing access to length scales pertinent to cell biology and monoatomic layer two-dimensional materials.

## Teaser

A new Hong-Ou-Mandel interferometer protocol achieves few-attosecond (nanometer) photon path delay resolution.

## INTRODUCTION

Since its discovery, Hong-Ou-Mandel (HOM) interferometry (*1*) has found a wide variety of applications within quantum optics (*2*–*7*). For example, it is commonly exploited as a measure of the distinguishability of photons produced by quantum dots (*8*, *9*). It can be used as a source of two-photon N00N states: a class of states widely studied in quantum metrology owing to their ability to reach the Heisenberg limit in phase-sensitive measurements (*10*–*14*). HOM interferometry is impervious to changes in the relative phase between the two photons, a property which implies that a HOM-based sensor does not require potentially impractical or expensive stabilization, as is typically required in classical interferometry. Furthermore, typical phase-dependent techniques suffer from a limited dynamic range equal to half of the wavelength due to the fact that multiple possible phases can correspond to the same signal. HOM interferometry, on the other hand, allows optical delays to be distinguished unambiguously over a range of a few micrometers owing to the relatively wide interference pattern (or HOM dip).

To date, the highest-precision time-delay measurements using the HOM effect make use of orthogonally polarized photon pairs to measure polarization mode dispersion (*15*, *16*). These studies have produced measurements of the group delay between pairs propagating along a common path to within a 0.1-fs uncertainty. The common-path geometry significantly aids the stability of the interferometer but can only be applied to (and thus is only relevant for) birefringent samples. A much wider range of applications is possible if the same or better precision can be achieved with a dual-arm geometry, which places no such restriction on how a sample might cause the relative delay.

Closely related to HOM interferometry, quantum optical coherence tomography (QOCT) is a method for extracting depth profiles of reflective interfaces, also via a HOM measurement of the relative delay between photon pairs, often implemented in a dual-arm geometry. In this context, features on the order of a micrometer have been detected, including those introduced by biological specimens (*17*–*20*). The limited depth resolution makes these approaches inadequate for smaller biological samples such as cell membranes, DNA samples, or protein monolayers, which have thicknesses on the order of 1 to 10 nm.

Both QOCT and standard HOM measurements have, to date, relied on detecting the shift in the interference minimum in the coincidence counts between the output ports of the interferometer.

Here, we devise and implement a completely new measurement and estimation strategy based on a Fisher information analysis. By tuning the interferometer to the delay that contains the maximum information content, and then by using a maximum-likelihood estimation (MLE) procedure, we achieve an improvement in precision and accuracy by two orders of magnitude over previous HOM approaches including QOCT. Throughout this work, we define the measurement precision as the SD of the final estimated photon delay, whereas the accuracy is defined as the difference between the final estimate and the known actual value. When both accuracy and resolution are good enough, it is possible to resolve small time delays (here on the order of a few attoseconds). We conducted measurements of the change in relative arrival time between two photons, δτ, with an average accuracy of 6 as (1.7 nm) and an average precision of 16 as (4.8 nm). Our best achieved accuracy was 0.5 as (0.15 nm) and best achieved precision was 4.7 as (0.9 nm). HOM interferometry can therefore enable single-photon characterization of optically transparent samples with thicknesses and length scales relevant, for example, to cell biology.

## RESULTS

**Fig. 1:**
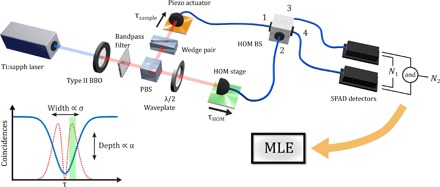
Dual-arm HOM interferometer. A pumped type II BBO crystal is used as a source of SPDC photon pairs, which are separated by a polarizing BS (PBS) before being subject to a differential time delay and recombination on separate input ports (1 and 2) of a fiber-coupled 50:50 BS (HOM BS). Coarse control of the optical delay is achieved by a motorized translation stage (HOM stage, controlling τ_HOM_), whereas fine control is achieved using a piezo actuator (controlling τ_sample_ and representing a transparent sample). From a scan of the HOM dip (bottom left, blue), a peak in the Fisher information (red) is identified to be used in the sensing procedure (green). The difference in temporal delay between the two photons (τ: = τ_sample_ − τ_HOM_) is quantified through MLE. Typical measured photon pair rates were on the order of 30,000 counts s^−1^ with an estimated loss rate of 87%. SPAD, single-photon avalanche photodiodes.

If two photons are incident on the input ports of a balanced beamsplitter (BS), the probability of a coincident detection at the output ports depends on the inner product of the quantum states of each photon and is influenced by the difference in arrival times of the two photons τ. HOM interference is characterized by the coincidence rate falling to zero as all distinguishing information is erased: the so-called “HOM dip” (see Fig. 1).

As Hong, Ou, and Mandel showed in their original work, if τ is scanned, the minimum position of the interference pattern can be measured with at least subpicosecond precision. Similar techniques were used in later works (*15*, *21*) and typically involve a simple least-squares fitting procedure for a scan over τ. By contrast, here, we use Fisher information analysis as the key theoretical tool for unlocking a peak-performance HOM interferometer. This allows us to introduce measurement and estimation protocols that are optimized to give a higher precision for a set amount of time invested or, equivalently, a greater information gain per photon.

The ultimate limit on the precision of estimation is known as the Cramér–Rao bound (*22*), which states that the variance of any unbiased estimator (that is, one whose expectation is equal to the true value of the parameter; see “Bias” section in Materials and Methods) must be bounded by

$$\text{Var}(\tilde{\tau })\geq \frac{1}{NF}$$ (1)

where $\tilde{\tau }$ denotes an estimator for the parameter τ. The Fisher information *F* measures the amount of information about τ that can be extracted from a particular experiment.

The HOM interferometer is characterized by many desirable features: for example, the large dynamic range (see the Supplementary Materials). Here, however, we are primarily concerned with minimizing $\text{Var}(\tilde{\tau })$. We achieve attosecond precision by accomplishing a combination of three goals: (i) maximizing *F*, (ii) saturating inequality (*1*), and (iii) increasing *N* (that is, the number of repetitions of the experiment) as much as possible within the confines of slow drift in the setup.

To achieve the first of our goals, we need to consider the dependence of *F* on τ and other parameters. Our statistical model is defined by a set of probabilities *P*_*i*_, where *i* = 0, 1, and 2 denotes the number of detectors that click in each run. The probabilities depend on the following parameters: the wave packet duration σ, which is proportional to the full width at half maximum of the temporal mode function of each photon (here taken to be Gaussian functions); the maximum indistinguishability α (which sets the visibility of the interference); and the photon loss rate γ (see Materials and Methods for the full model). The Fisher information evaluates to

$$F=\frac{1}{\sigma^{2}}(\frac{{2\alpha }^{2}\tau^{2}e^{-2\tau^{2}/\sigma^{2}}}{\frac{\left(1-{\alpha e}^{-\tau^{2}/\sigma^{2}}\right)}{{\left(1-\gamma \right)}^{2}}-\frac{1}{2}{\left(1-{\alpha e}^{-\tau^{2}/\sigma^{2}}\right)}^{2}})$$ (2)

**Fig. 2:**
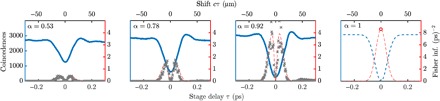
Experimental HOM dips (left axis) are shown for various visibilities, introduced by a differential polarization change. The estimated total Fisher information *NF* (Eq. 2; right axis, red) along with the inverse variance of our experimental estimates are shown (gray crosses, right axis). The rightmost panel includes theoretical curves for perfect visibility, where the two peaks in the Fisher information have asymptotically merged. The open circle denotes a point where the Fisher information is undefined.

The distribution of information in Eq. 2 is doubly peaked and symmetric around τ = 0 (see red dashed curves in Figs. 1 and 2). We note that when α → 1 (perfect visibility), the peaks asymptotically merge at the origin. Because the visibility is reduced (as it is in all experiments), the maximum information decreases and the peaks move outward. A similar phenomenon was observed in the study of Jachura *et al*. (*23*), where nonunit visibility caused a marked qualitative change in the distribution of Fisher information for a phase-sensitive interferometer. Here, the changing distribution reflects the competition between (i) the derivative of the inverted Gaussian dip (which is optimized at $\tau =\sigma /\sqrt{2}$) and (ii) the variance of *P*_*i*_ (which is optimized at τ = 0). This suggests that using prior knowledge of τ and α enables the interferometer to be tuned to operate at the optimum point between these extremes. In Materials and Methods, we discuss how we found this point.

To achieve our second goal of saturating the Cramèr-Rao bound, we use the MLE

$$\tilde{\tau }=\pm \sigma \sqrt{\text{ln}(\frac{\alpha (N_{1}+N_{2})}{N_{1}-N_{2}(\frac{1+3\gamma }{1-\gamma })})}$$ (3)

where *N*_1_ (respectively *N*_2_) is the number of times only one (both) detector(s) clicked (see Materials and Methods). This estimator is efficient, that is, it saturates Eq. 1 when the number of trials is large enough (*24*). When the argument of the logarithm is negative, the likelihood is maximized at τ → ±∞. To account for this situation (which arises when the data set is very noisy), we use a Bayesian analysis (see Materials and Methods). The estimator is nonlinear but analytically calculable and thus suitable for real-time estimation with the data. Note that due to the symmetry of the HOM dip, there is a twofold ambiguity in the estimate—we can only obtain its magnitude and not its sign. As we shall see later, this issue will be resolved through our measurement protocol. Note that the estimation involves observable quantities *N*_*i*_, as well as γ, σ, and α. The latter need to be separately estimated before the measurements begin (see Materials and Methods).

To test our theory and demonstrate our protocol, a noncommon-path HOM interferometer was constructed using spontaneous parametric down-conversion (SPDC) from a type II nonlinear crystal as a source of orthogonally polarized photon pairs (see Fig. 1 and Materials and Methods). The photons are deterministically separated via their polarization, and each is collected by a single-mode fiber coupled to a BS (HOM BS, Fig. 1). The relative path-length difference between the photon pair was controlled with a coarse stage.

The HOM dip was scanned using a 10-nm bandpass filter positioned before the PBS to ensure spectral indistinguishability and therefore high-visibility two-photon interference. As a first step, a total of 50 scans of the HOM dip were acquired to ascertain the variance of the estimates and compare with the predicted Fisher information (which acts as an upper bound on the inverse variance). To test the theoretical model further, the polarization of one of the arms of the interferometer is rotated so as to reduce the visibility of the interference to approximately 50%. The inverse variance of our estimates follows the predicted Fisher information distribution, as shown in Fig. 2.

These data therefore constitute a good confirmation of Eqs. 1 and 2. By replacing the interference filter, it was observed that both the measurement accuracy and precision were improved for wider bandwidth photons: This is despite of the reduction in α and due to the decrease in σ and increase in *N*. For this reason, the bandpass filter was removed and replaced with a long-pass edge filter to block the pump field of the SPDC without altering the spectrum of signal and idler.

For subsequent measurements, we introduced attosecond-scale temporal delays, τ_sample_ (see Fig. 1), with an additional piezo actuator (thus playing the role of a transparent sample that can be inserted in and out of the photon path). The HOM interferometer delay (τ_HOM_) was first tuned with a coarse control stage to a maximum in the Fisher information (see Fig. 2) and was kept there while a large amount of data [*O*(10^9^) counts] was collected.

To achieve our third goal (increasing the number of measurements within the limits of experimental drift), the piezo actuator was periodically switched (every 100 ms) between the two positions (which we label “in” and “out”), and we collected a set of counts *N*_*i*_ corresponding to each. We then use this data (in combination with the parameters *N*_*i*_, γ, σ, and α) to estimate τ^in^ and τ^out^. Finally, we extract the difference in differential time delay $\delta \tau :=\tau^{\text{in}}-\tau^{\text{out}}=(\tau_{\text{sample}}^{\text{in}}-\tau_{\text{HOM}})-(\tau_{\text{sample}}^{\text{out}}-\tau_{\text{HOM}})=\tau_{\text{sample}}^{\text{in}}-\tau_{\text{sample}}^{\text{out}}$. From prior knowledge of the system, we make the assumption that the sign of $\tau_{\text{sample}}^{\text{in}}$ and $\tau_{\text{sample}}^{\text{out}}$ are the same such that the value of δτ is unambiguous.

**Fig. 3:**
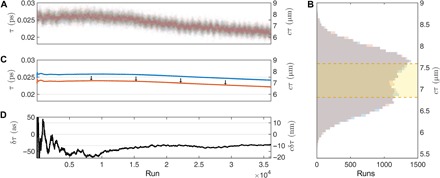
Example of an acquired experimental data set. (**A**) Individual estimates of τ^in^ (blue) and τ^out^ (red) for two piezo positions separated by 10 nm (33.3 as). A relatively large drift can be seen in the data, which is dealt with by switching between piezo positions as discussed above. (**B**) The histogram shows two almost perfectly overlapping distributions. Classical interferometry would be limited to a distribution no larger than the yellow area. The distributions are generally nonunimodal, which is indicative of significant drift (or slowly varying noise). (**C**) Cumulative estimates are plotted. The drift in each estimate is considerable, being approximately 2 fs (600 nm). The red curve has been shifted down by 2 fs for clarity (arrows). The drift for each sample position is very well correlated because we switch the sample position much faster than the drift. (**D**) Because of this correlation, the difference in cumulative estimates δτ is very stable and converges on the true value.

Figure 3 shows data for a sample position separation (*cδτ*) of 10 nm. Each individual integration window yields a relatively poor precision $\sqrt{\text{Var}(\tilde{\delta }\tau )}=\sqrt{\text{Var}({\tilde{\tau }}^{\text{in}})+\text{Var}({\tilde{\tau }}^{\text{out}})}\approx 600$ as (180 nm) estimate of the optical delay. Figure 3 also shows cumulative estimates, which correspond to a single data set which is gradually incremented, accumulating all the counts of individual data sets (which are assumed independent). Initially, a cumulative data set (and estimate) will change rapidly before settling down later on. By assuming that each experiment is independent, we can combine *M* = *O*(10^4^) such data sets to achieve a *O*(100)-fold improvement in precision, that is, toward a few attoseconds (few nanometers). The precision obtained using the entire data set is estimated as $\sqrt{\text{Var}(\delta \tilde{\tau })/M}$. Figure 3B shows that the distribution of the individual measurements in our approach falls well outside the λ/2 (404 nm, highlighted in yellow) range that is acceptable for phase-dependent interference methods. Despite this, our approach still recovers accurate estimates, and its dynamic range is determined by the size of the HOM dip (see Fig. 2): Up to an ambiguity in the sign of τ, there is a clear one-to-one mapping between τ (which we estimate) and the coincidences (which we measure) over a range of at least 25 μm.

**Fig. 4:**
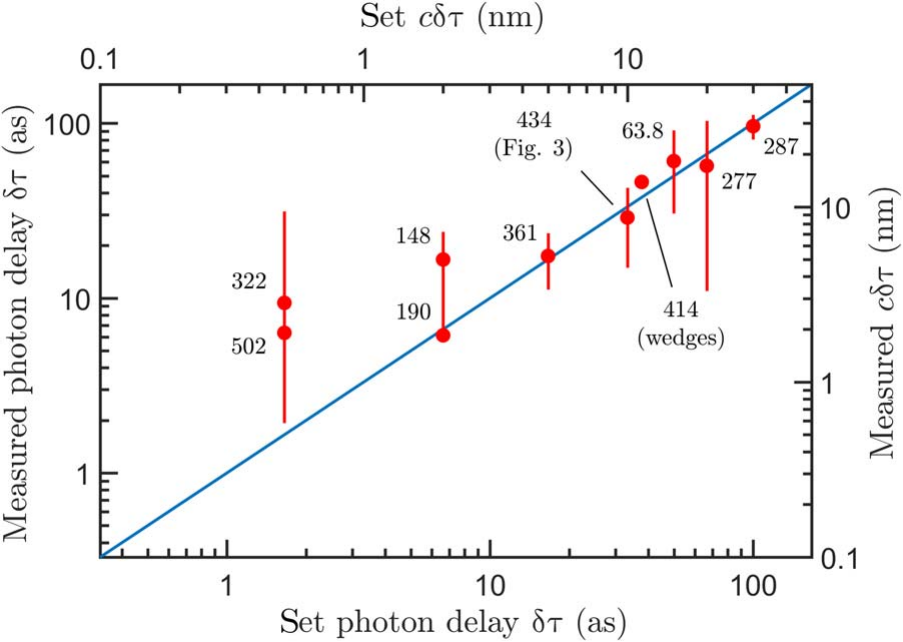
Experimentally measured photon delays induced by the piezo shown against the set values on the piezo actuator. Number of individual measurements and integrations times vary as indicated in the plot (labels denote billions of incident biphotons). Total acquisition times for each data point ranged between 1.4 and 15.6 hours. Error bars represent an interval of length $2\sqrt{\text{Var}(\tilde{t})}$. The data point corresponding to the glass wedges should only be read on the top and right axes (because of the nonunit refractive index).

Next, a series of target piezo displacements (τ_sample_) were set to test the capabilities of our protocol. Figure 4 shows the final estimated shifts compared to the ground truth displacements recorded by the internal capacitive sensor of the piezo actuator. The measurement procedure consistently returns a high degree of accuracy even down to the smallest set displacements of 1.5 as (0.5 nm): Typical values of the measurement precision are within the ±6 to 15–as (±2 to 5 nm) range.

We performed a final experiment to demonstrate the potential for our scheme to measure samples scanned transversely across the photon path and thus perform full imaging tasks. We introduce a controlled delay using a pair of transparent wedges positioned in one of the interferometer arms resulting in an asymmetric loss of around 30%. The wedges are arranged such that translating one of the wedges changes the length of propagation through the glass while maintaining the alignment of the system (as shown in Fig. 1). A target delay of 57 as, resulting from an estimated 11 nm of additional glass, was introduced using the wedge pair (taking the refractive index of glass to be 1.5). Our measurement procedure returned a measured delay of 69 ± 5 as, giving an estimate of 14 ± 1 nm for the additional glass length. We attribute the difference between the measured and expected values to an imperfect calibration of the wedge system.

## DISCUSSION

If we compare our results to the literature, the best HOM measurement performed to date had an accuracy of 200 as (60 nm) and a precision of 100 as (30 nm) (*15*) using a common-path interferometer geometry. We show an accuracy improvement of approximately 31× with neither the benefit of the inherent stability nor the limitation to birefringent samples. If compared to noncommon-path HOM interferometers, previous work showed resolutions on the order of a few femtoseconds (approximately micrometers), with respect to which we have a more than 100× improvement (*17*–*21*). Our technique opens for the first time the possibility of using HOM interference to perform measurements of transparent samples in the single-attosecond delay (that is, subnanometer path length) regime. Scan-free imaging capability could also be potentially introduced by resorting to wide-field lensless approaches recently demonstrated with a classical interferometer (*25*). With a small modification, the technique can also be applied to reflective samples such as those used in QOCT experiments providing the same enhancement to the precision. Furthermore, there is a significant scope to increase the precision of our experiment yet further through the use of shorter down-conversion crystals (lower σ) and/or higher-efficiency photodetectors (lower γ) (*16*). For example, combining our method with the engineered source of photon pairs from the work of Okano *et al*. (*26*) has the potential to yield another 30-fold improvement in precision arising from the increased bandwidth of the photon pairs. The HOM dip can also be specifically tailored to further optimize the amount of Fisher information obtainable. By using the best available photodetectors with upward of 95% efficiency (γ = 0.05) (*27*) and using Eq. 2, we estimate that (holding α constant at 0.9) one could achieve an approximate 50× improvement in the Fisher information or around 7× improvement in precision. Equally, an increase in the Fisher information allows the total acquisition time for each measurement to be reduced while maintaining the same precision. With a 50× improvement in the Fisher information, the longest duration measurement presented here could be reduced to < 20 min.

Finally, we note that our interferometer is capable of producing phase-sensitive fringes (as shown in the Supplementary Materials) by rotating the PBS away from being perfectly aligned with the signal and idler polarization reference frame (*28*). Here, these fringes that are due to *N* = 2 N00N state interference have been suppressed to investigate the attainable precision using two-photon interference alone. In the future, however, it would be possible to further increase the Fisher information by simply rotating the input photon polarization (see the Supplementary Materials for details). As is also the case for classical interferometry, such an approach would require the use of active phase stabilization, or switching between sample positions 100× faster than we did above in our inherently phase-insensitive approach. This would nevertheless allow for a further 150-fold improvement in precision, allowing measurements to reach into the picometer length scale.

## MATERIALS AND METHODS

### Theory

#### Quantum mechanical derivation of the HOM effect

Assume that we have a source that can produce two-photon states of the form

$$|\psi \rangle =(\eta [a_{1}^{†}a_{2}^{†}]+\sqrt{1-{\left|\eta \right|}^{2}}[b_{1}^{†}a_{2}^{†}])|\text{vac}\rangle$$ (4)

where the operator $a_{i}^{†}$ creates a photon with certain properties (frequency distribution, polarization, and so on) in mode *i* = 1 and 2 corresponding to the two input modes of a balanced BS (see Fig. 1), $b_{i}^{†}$ creates a photon in an orthogonal mode (say with orthogonal polarization) and |vac〉 is the vacuum. η is a real parameter describing the degree of overlap of the quantum states in modes 1 and 2. Because reflection at the BS requires a phase shift of 90°, we represented the BS transformation using the conventions

$$\begin{array}{c} a_{1}^{†}\to (ia_{3}^{†}+a_{4}^{†})/\sqrt{2}\\ a_{2}^{†}\to (a_{3}^{†}+ia_{4}^{†})/\sqrt{2} \end{array}$$ (5)

and similarly for the *b* modes. The indices 3 and 4 denote the output ports of the BS. Then, one has

$$\begin{array}{c} |\psi \rangle \to \frac{1}{2}(\eta [ia_{3}^{†}a_{3}^{†}+\overset{\overset{=0}{⏞}}{a_{4}^{†}a_{3}^{†}-a_{3}^{†}a_{4}^{†}}+ia_{4}^{†}a_{4}^{†}]+\\ \sqrt{1-{\left|\eta \right|}^{2}}[ib_{3}^{†}a_{3}^{†}+b_{4}^{†}a_{3}^{†}-b_{3}^{†}a_{4}^{†}+ib_{4}^{†}a_{4}^{†}])|\text{vac}\rangle \end{array}$$ (6)

The cancellation above is a consequence of the bosonic commutation relation $[a_{3}^{†},a_{4}^{†}]=0$. Now assuming that we have detectors that indiscriminately register coincidences (one photon in mode 3 and one photon in mode 4), the probability of this occurring is

$$P_{c}=\frac{1}{2}(1-{\left|\eta \right|}^{2})$$ (7)

Expanding the signal and idler photons into an orthonormal time-bin basis $\langle t|t^{\prime }\rangle =\delta_{tt^{\prime }}$

$$\begin{array}{c} \eta (\tau )=\langle \text{vac}|(\eta a_{1}+\sqrt{1-{\left|\eta \right|}^{2}}b_{1})a_{2}^{†}|\text{vac}\rangle =\langle \text{signal}|\text{idler}\rangle \\ =\sqrt{\alpha }\langle t|\int \int f_{1}^{*}(t-\tau_{\text{sample}})f_{2}(t\prime -\tau_{\text{HOM}})dtdt\prime |t\prime \rangle \\ =\sqrt{\alpha }\int f_{1}^{*}(t-\tau_{\text{sample}})f_{2}(t-\tau_{\text{HOM}})dt\\ =\sqrt{\alpha }\int f_{1}^{*}(t-\tau )f_{2}(t)dt \end{array}$$ (8)

where we defined τ = τ_sample_ − τ_HOM_. Here, *f*_*i*_ is the temporal mode function of the photon in each input port of the BS. The time-delay τ (which transforms *a*_1_ toward *b*_1_) was introduced either through a controllable translation stage or a transparent sample of unknown refractive properties. α is a positive phenomenological parameter representing residual distinguishability for perfectly synchronized modes, contributed to by polarization, spatial mode, or other mismatches, as well as any imbalance in the BS. The temporal mode functions were set by the longitudinal uncertainty in the location of the down-conversion event. When this is limited by the length of the crystal, the mode functions are top-hat functions, leading to a triangular dip. Here, we assumed that the photons have Gaussian temporal mode functions [in our case, they do so natively: Otherwise, commonly used spectral filters can be used to broaden and reshape the temporal distribution, leading once again to a Gaussian dip (*16*)]. If *f*_1_ = *f*_2_ and are both Gaussians with SD $\sigma /\sqrt{8}$, then we obtain

$$P_{c}=\frac{1}{2}(1-\alpha e^{-s^{2}})$$ (9)

where we defined the normalized temporal delay *s* = τ/σ, which is a dimensionless quantity.

#### Loss model

Real experiments are subject to losses—in our case, they were dominated by the inefficiency of our photodetectors. We therefore modeled this by allowing for a photon to be lost with probability γ immediately before detection. The full model is thus given by

$$\left(\begin{array}{c} P_{0}\\ P_{1}\\ P_{2} \end{array})=(\begin{array}{cc} \gamma^{2} & \gamma^{2}\\ 2\gamma (1-\gamma ) & 1-\gamma^{2}\\ 1-2\gamma (1-\gamma )-\gamma^{2} & 0 \end{array})(\begin{array}{c} P_{c}\\ P_{b} \end{array}\right)$$ (10)

with *P*_*b*_ = 1 − *P*_*c*_ implied by normalization. By transforming Eq. 9, the resultant model is

$$P_{2}=\frac{1}{2}{\left(1-\gamma \right)}^{2}(1-\alpha e^{-s^{2}})$$ (11)

$$P_{1}=\frac{1}{2}{\left(1-\gamma \right)}^{2}(\frac{1+3\gamma }{1-\gamma }+\alpha e^{-s^{2}})$$ (12)

$$P_{0}=\gamma^{2}$$ (13)

We have used the label *i* = 0, 1, and 2 to denote the number of detectors that click—that is, a total loss, bunch, and coincidence, respectively. The total number of incident photon pairs is given by *N* = *N*_0_ + *N*_1_ + *N*_2_. Note that *N*_0_ is a purely theoretical quantity used to define our model and need not (and in fact cannot) be measured at all.

#### Fisher information

The Fisher information *F*_*s*_ is defined as a functional of a statistical model *P*(*i*|*s*), which is a normalized set of probabilities for outcomes *i* conditioned on the value of our target parameter *s* (such as those above). The Fisher information in the main text may be calculated as

$$F_{\tau }=\frac{1}{\sigma^{2}}F_{s}=\frac{1}{\sigma^{2}}\sum_{i}\frac{{\left(\partial_{s}P(i|s)\right)}^{2}}{P(i|s)}$$ (14)

#### Maximum-likelihood estimator

The likelihood is a multinomial distribution $L(N_{1},N_{2}|\tau )\propto P_{0}^{N_{0}}P_{1}^{N_{1}}P_{2}^{N_{2}}$ (where the constant of proportionality does not depend on *s*). We extremized the likelihood as follows

$$\begin{array}{c} 0=:{\left(\partial_{s}\text{log}L\right)}_{{\tilde{s}}_{\text{MLE}}}\\ =\partial_{s}(N_{0}\text{log}(P_{0}))\\ +\partial_{s}{\left(N_{1}\text{log}(P_{1})\right)}_{{\tilde{s}}_{\text{MLE}}}+\partial_{s}{\left(N_{2}\text{log}(P_{2})\right)}_{{\tilde{s}}_{\text{MLE}}}\\ ={\frac{N_{1}P_{1}^{\prime }}{P_{1}}|}_{{\tilde{s}}_{\text{MLE}}}+{\frac{N_{2}P_{2}^{\prime }}{P_{2}}|}_{{\tilde{s}}_{\text{MLE}}}\\ ={\frac{N_{1}P_{1}^{\prime }}{P_{1}}|}_{{\tilde{s}}_{\text{MLE}}}-{\frac{N_{2}P_{1}^{\prime }}{P_{2}}|}_{{\tilde{s}}_{\text{MLE}}}\\ {N_{1}P_{2}|}_{{\tilde{s}}_{\text{MLE}}}={N_{2}P_{1}|}_{{\tilde{s}}_{\text{MLE}}} \end{array}$$ (15)

This equation is then solved for ${\tilde{s}}_{\text{MLE}}$. We discarded the minimum-likelihood solution at *s* = 0. The solution, which corresponds to a maximum, was described in the main text.

#### Peak information point

When α = 1, the Fisher information given in the main text was maximized near *s* = 0, although it is undefined there. We have

$$\underset{s\to 0}{\text{lim}}F_{s}{|}_{\alpha =1}=2$$

**Fig. 5:**
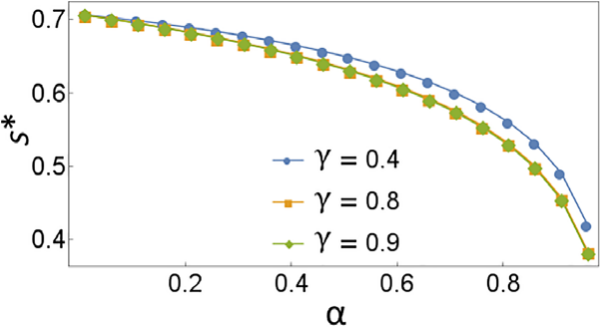
Optimal sensitivity point as a function of visibility. The three curves correspond to different values of the photon loss rate **γ**.

As α is lowered, two true peaks appeared, moving outward and becoming broader. When γ = 0, we have

$$F_{s}=\frac{4\alpha^{2}s^{2}}{e^{2s^{2}}-\alpha^{2}}$$

with maximum

$$s*=\frac{\pm \sqrt{W(-\frac{\alpha^{2}}{e})+1}}{\sqrt{2}}$$

for *W* the Lambert *W* function. When γ ≠ 0, s* can be found numerically (it has a rather weak dependence on γ). For α → 0, the optimum moves to the inflection point of the Gaussian, $s*\to \pm 1/\sqrt{2}$ (see Fig. 5).

#### Bayesian analysis

To avoid infinities, we used a prior distribution *p*(*s*) uniform on [−*s*_max_, *s*_max_]. Instead of directly maximizing the likelihood, we instead used it to multiplicatively update the prior distribution before maximizing the resultant posterior distribution. This is an application of the Bayes rule

$$p(s|N_{1},N_{2})\propto L(N_{1},N_{2}|s)p(s)$$ (16)

(again, the proportionality constant does not depend on *s*). The maximum of this posterior is unchanged (with respect to the likelihood) when the argument of the logarithm in Eq. 3 is positive. When the argument is not positive, the maximum posterior is at *s* = ±*s*_max_. So our full, maximum a posteriori estimator is

$${\tilde{s}}_{\text{MAP}}=\{\begin{array}{cc} \tilde{s} & \text{for}\;N_{1}-N_{2}(\frac{1+3\gamma }{1-\gamma })>0\\ \pm s_{\text{max}} & \text{for}\;N_{1}-N_{2}(\frac{1+3\gamma }{1-\gamma })\leq 0 \end{array}$$ (17)

We set *s*_max_ = 10. We also have $\tilde{\tau }=\sigma \tilde{s}$ where estimators are denoted with a ~.

#### Calibration stage

Our measurement protocol comprises several steps beginning with a full calibration of the parameters *N*, γ, σ, and α. First, the interferometer was tuned far outside the dip, and we calculated the photon loss parameter γ

$$\tilde{\gamma }={\frac{N_{1}-N_{2}}{N_{1}+3N_{2}}|}_{s\to \infty }$$ (18)

Next, to allow us to estimate the precision of our experiment, we also estimated the total number of incident photon pairs

$$\tilde{N}=\frac{N_{1}+N_{2}}{1-\gamma^{2}}$$ (19)

Now, we varied τ_HOM_ to perform a partial scan of the dip that covers both the *s* = 0 and *s* = s* points.

Then, we have

$$\tilde{\alpha }=1-\frac{2\text{min}(N_{2})}{N{\left(\gamma -1\right)}^{2}}$$ (20)

Finally, we applied $\tilde{s}$ to the partial scan of the dip. This irons out the bell-shaped dip to a roughly linear V-shape (see the Supplementary Materials). We then performed a linear fit near the target region, and σ was taken as the inverse of the gradient. We chose the size of the fitting window to be approximately 7 fs (see the Supplementary Materials).

#### Procedure for the local fitting of the HOM dip

**Fig. 6:**
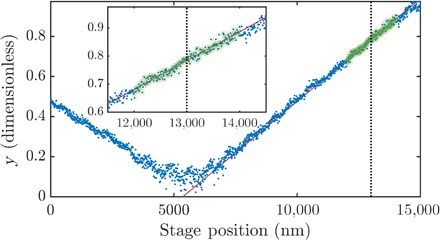
Representative example of our fitting procedure to extract σ during the calibration of the HOM dip. Inset: Enlarged view of the region of interest for the sensing procedure.

The width of the HOM dip is the final fit parameter to be estimated. To construct an estimate, we performed a partial scan of the HOM dip, which results in a list of triples (τ, *N*_1_, and *N*_2_), where τ is the “ground truth” optical delay inferred from electronic readout of the piezo stage. Using the already estimated values of α and γ, we reduced each triple using the estimator $\tilde{s}$ (which is nothing other than $\tilde{\tau }/\sigma$; see Eq. 3). This estimator is a function of *N*_1_ and *N*_2_ and maps the list of triples into a list of pairs (τ and *s*). This has the effect of straightening the Gaussian dip into a “vee,” as seen in Fig. 6. Because τ = σ*s*, we can perform a linear fit of these data. We chose to perform the fit in a restricted region about 7 fs wide, centered on the point of maximum Fisher information. Our estimate of σ is simply the inverse of the gradient: $\tilde{\sigma }=\Delta \tau /\Delta s$.

#### Bias

Because the dominant sources of imperfection were accounted for in our model, we expected the measured precision (related to the inverse root of the Fisher information) and measured accuracy to closely match the theoretical quantities, although small discrepancies were to be expected because of uncontrollable sources of random and systematic errors. The full Cramér-Rao bound is

$$\text{Var}(\tilde{\tau })\geq \frac{{\left(1+\frac{\partial b(\tau )}{\partial \tau }\right)}^{2}}{NF}\approx \frac{1}{NF}$$ (21)

with $b(\tau )=E(\tilde{\tau }-\tau )$ being the bias (E is the expected value). The MLE is consistent, meaning that the bias is zero in the limit of *N* → ∞ (*29*). Because we have a very large *N*, the bias should therefore be negligible.

### Experiments

A frequency-doubled Ti:sapphire oscillator (Coherent Chameleon Ultra II) with a 130-fs duration at a repetition rate of 80 MHz was used to pump a 0.5-mm-long type II BBO crystal for wavelength-degenerate SPDC. The 808-nm signal and idler photons were spatially separated using a PBS and then coupled into polarization-maintaining fibers where they were guided to a fiber-coupled 50:50 cube BS (HOM BS), as shown in Fig. 1. Coarse control of the interferometer delay, τ_HOM_, was controlled by adjusting the on-axis position of one of the fiber couplers using a translation stage (HOM stage). In this manner, the HOM dip can be characterized by counting coincident events between two single-photon avalanche photodiode detectors, which were positioned at the output arms of the HOM BS because the delay was changed. Timing for the coincident event detection was managed by an event timing module (Picoquant HydraHarp 400). Fine control of the delay was achieved by moving the other fiber coupler with a piezo actuator controlled translation stage (piezo actuator, PI P-753.1CD). This configuration allowed for precise control of the optical path length with a subnanometer resolution.

The translating wedge system was calibrated by removing the spectral filters prohibiting the second harmonic generation pump beam from reaching the BBO used for down-conversion and allowing the coherent state of the laser at 808 nm to pass through the setup as a Mach-Zehnder interferometer. The beam was attenuated to the single-photon level and a half waveplate before the PBS was rotated to balance the photon count level in each interferometer arm to yield high-visibility interference. The period of the resulting interference fringes allows us to define a conversion factor of a 1-μm translation of the wedges result in an effective path length change of approximately 17 nm.

## Supplementary Material

http://advances.sciencemag.org/cgi/content/full/4/5/eaap9416/DC1

## SUPPLEMENTARY MATERIALS

Supplementary material for this article is available at http://advances.sciencemag.org/cgi/content/full/4/5/eaap9416/DC1 Dynamic range of the measurement procedure Activating phase fringes List of fitting parameters and results fig. S1. Predicted Fisher information for a HOM with added phase-dependent fringes. table S1. Summary of all measurements and parameters used in the fitting procedure.
