# Supplementary material for: Attosecond-resolution Hong-Ou-Mandel interferometry
Source: Sci Adv. 2018 May 4;4(5):eaap9416. doi: 10.1126/sciadv.aap9416 (PMC5935478; doi:10.1126/sciadv.aap9416)
Supplement: http://advances.sciencemag.org/cgi/content/full/4/5/eaap9416/DC1 [file supp_4_5_eaap9416__index.html]

Science Advances | Science Advances

## Supplementary Materials

**This PDF file includes:**

- Dynamic range of the measurement procedure
- Activating phase fringes
- List of fitting parameters and results
- fig. S1. Predicted Fisher information for a HOM with added phase-dependent fringes.
- table S1. Summary of all measurements and parameters used in the fitting procedure.

Download PDF

**Files in this Data Supplement:**

- Adobe PDF - aap9416\_SM.pdf
