# Supplementary material for: Attosecond-resolution Hong-Ou-Mandel interferometry
Source: Sci Adv. 2018 May 4;4(5):eaap9416. doi: 10.1126/sciadv.aap9416 (PMC5935478; doi:10.1126/sciadv.aap9416)
Supplement: http://advances.sciencemag.org/cgi/content/full/4/5/eaap9416/DC1 [file aap9416_SM.pdf]

Science Advances

AAAS

advances.sciencemag.org/cgi/content/full/4/5/eaap9416/DC1

Supplementary Materials for

Attosecond-resolution Hong-Ou-Mandel interferometry

Ashley Lyons, George C. Knee, Eliot Bolduc, Thomas Roger, Jonathan Leach, Erik M. Gauger, Daniele Faccio

Published 4 May 2018, *Sci. Adv.* **4**, eaap9416 (2018)
DOI: 10.1126/sciadv.aap9416

This PDF file includes:

- Dynamic range of the measurement procedure
- Activating phase fringes
- List of fitting parameters and results
- fig. S1. Predicted Fisher information for a HOM with added phase-dependent fringes.
- table S1. Summary of all measurements and parameters used in the fitting procedure.

Dynamic range of the measurement procedure

The dynamic range may be defined as the maximum interval in τ such that the estimator is single-valued. Phase sensitive interferometers (such as the Mach-Zehnder) suffer from a 'phase-wrapping' problem, where a multitude of physical time delays result in the same relative phase in the interferometer. Those physical time delays cannot then be mutually distinguished, unless some prior information is available. The dynamic range of a Mach-Zehnder interferometer is set by the wavelength of light that is used. One can use a uniform prior distribution to exclude parameters outside a certain interval: this can restore the uniqueness of estimates, at the expense of having enough prior information (represented by the inverse width of the interval). A higher dynamic range implies that less prior information is required to avoid ambiguities. In principle the dynamic range of our Gaussian HOM interferometer is infinite because of the long tails of $f(t)$. In practice, however, one should cap the dynamic range to a region where F is above some threshold value. A conservative estimate would be on the order of 1 or 2 σ, which is typically 10-100$\times$ larger than the wavelength of light in a phase dependent (e.g. MZ) interferometer.

Activating phase fringes

As we alluded to in the main text, it is straightforward for our setup to be converted to a phase sensitive one by including a single optical element ($\lambda/2$ waveplate before the PBS). Here we will show the full effect of an arbitrary rotation of the waveplate on the coincidence probability, and the significantly increased Fisher information that results.

Consider misalignment between the downconversion crystal and the PBS. Assume that horizontal (H) and vertical (V) polarisations are defined by the PBS, and that the downconversion source is rotated around the pump propagation axis by an angle θ. Let us model the SPDC state as:

$$|\psi\rangle_{\mathrm{SPDC}} = \Big[\eta(\cos(\theta)a_{1H}^\dagger + \sin(\theta)a_{1V}^\dagger)(\sin(\theta)a_{1H}^\dagger - \cos(\theta)a_{1V}^\dagger) + \beta(\cos(\theta)b_{1H}^\dagger + \sin(\theta)b_{1V}^\dagger)(\sin(\theta)a_{1H}^\dagger - \cos(\theta)a_{1V}^\dagger)\Big]|\mathrm{vac}\rangle \quad (1)$$

where the creation operators now carry polarisation labels. The PBS has the action of transforming V photons to mode 2 with a phase shift of i due to reflection. It has no effect on H photons:

$$|\psi\rangle_{\mathrm{PBS}} = \Big[\eta(\cos(\theta)a_{1H}^\dagger + i\sin(\theta)a_{2V}^\dagger)(\sin(\theta)a_{1H}^\dagger - i\cos(\theta)a_{2V}^\dagger) + \beta(\cos(\theta)b_{1H}^\dagger + i\sin(\theta)b_{2V}^\dagger)(\sin(\theta)a_{1H}^\dagger - i\cos(\theta)a_{2V}^\dagger)\Big]|\mathrm{vac}\rangle. \quad (2)$$

Next the HWP takes V to H and H to V but only in mode 2 (this should also be well aligned with the PBS):

$$\begin{aligned}|\psi\rangle_{\mathrm{HWP}} &= \Big[\eta(\cos(\theta)a_{1H}^\dagger + i\sin(\theta)a_{2H}^\dagger)(\sin(\theta)a_{1H}^\dagger - i\cos(\theta)a_{2H}^\dagger) + \beta(\cos(\theta)b_{1H}^\dagger + i\sin(\theta)b_{2H}^\dagger)(\sin(\theta)a_{1H}^\dagger - i\cos(\theta)a_{2H}^\dagger)\Big]|\mathrm{vac}\rangle \\ &= \Big[\eta(\cos(\theta)a_1^\dagger + i\sin(\theta)a_2^\dagger)(\sin(\theta)a_1^\dagger - i\cos(\theta)a_2^\dagger) + \beta(\cos(\theta)b_1^\dagger + i\sin(\theta)b_2^\dagger)(\sin(\theta)a_1^\dagger - i\cos(\theta)a_2^\dagger)\Big]|\mathrm{vac}\rangle. \end{aligned} \quad (3)$$

We now drop the polarization labels since all photons have the same value now. Next we introduce the path length change of τ, which gives rise to the phase shift $e^{i\phi} = e^{i2\pi\tau/\lambda}$ in mode 2.

$$|\psi\rangle_{\mathrm{shift}} = \Big[\eta(\cos(\theta)a_1^\dagger + ie^{i\phi}\sin(\theta)a_2^\dagger)(\sin(\theta)a_1^\dagger - ie^{i\phi}\cos(\theta)a_2^\dagger) + \beta(\cos(\theta)b_1^\dagger + ie^{i\phi}\sin(\theta)b_2^\dagger)(\sin(\theta)a_1^\dagger - ie^{i\phi}\cos(\theta)a_2^\dagger)\Big]|\mathrm{vac}\rangle. \quad (4)$$

Now the final step is the beamsplitter (whose action is shown in the main text),

$$\begin{aligned}
|\psi\rangle_{\mathrm{BS}} = \Big[&\frac{\eta}{2}(\cos(\theta)[ia_3^\dagger + a_4^\dagger] + ie^{i\phi}\sin(\theta)[a_3^\dagger + ia_4^\dagger])(\sin(\theta)[ia_3^\dagger + a_4^\dagger] - ie^{i\phi}\cos(\theta)[a_3^\dagger + ia_4^\dagger]) \\
&+ \frac{\beta}{2}(\cos(\theta)[ib_3^\dagger + b_4^\dagger] + ie^{i\phi}\sin(\theta)[b_3^\dagger + ib_4^\dagger])(\sin(\theta)[ia_3^\dagger + a_4^\dagger] - ie^{i\phi}\cos(\theta)[a_3^\dagger + ia_4^\dagger])\Big]|\mathrm{vac}\rangle \\
= \Big[&\frac{\eta}{2}[a_3^\dagger(i\cos(\theta) + ie^{i\phi}\sin(\theta)) + a_4^\dagger(\cos(\theta) - e^{i\phi}\sin(\theta))][a_3^\dagger(i\sin(\theta) - ie^{i\phi}\cos(\theta)) + a_4^\dagger(\sin(\theta) + e^{i\phi}\cos(\theta))] \\
&+ \frac{\beta}{2}[b_3^\dagger(i\cos(\theta) + ie^{i\phi}\sin(\theta)) + b_4^\dagger(\cos(\theta) - e^{i\phi}\sin(\theta))][a_3^\dagger(i\sin(\theta) - ie^{i\phi}\cos(\theta)) + a_4^\dagger(\sin(\theta) + e^{i\phi}\cos(\theta))]\Big]|\mathrm{vac}\rangle.
\end{aligned} \tag{5}$$

The next step is to combine the correct amplitudes to get a coincidence probability. There are amplitudes for $a_3^\dagger a_4^\dagger$ and $a_4^\dagger a_3^\dagger$:

$$\begin{aligned}
\mathcal{A}_{a_3^\dagger a_4^\dagger} &= \frac{\eta}{2}\Big[(i\cos(\theta) + ie^{i\phi}\sin(\theta))(\sin(\theta) + e^{i\phi}\cos(\theta))\Big], \\
\mathcal{A}_{a_4^\dagger a_3^\dagger} &= \frac{\eta}{2}\Big[(\cos(\theta) - e^{i\phi}\sin(\theta))(i\sin(\theta) - ie^{i\phi}\cos(\theta))\Big].
\end{aligned} \tag{6}$$

These operators commute, because of the orthogonal spatial modes: therefore $a_3^\dagger a_4^\dagger = a_4^\dagger a_3^\dagger$ and we should add these terms coherently, allowing the HOM interference effect to operate. There are two more amplitudes (that cannot interfere) for $b_3^\dagger a_4^\dagger$ and $b_4^\dagger a_3^\dagger$ (where once again operators commute):

$$\begin{aligned}
\mathcal{A}_{b_3^\dagger a_4^\dagger} &= \frac{\beta}{2}\Big[(i\cos(\theta) + ie^{i\phi}\sin(\theta))(\sin(\theta) + e^{i\phi}\cos(\theta))\Big], \\
\mathcal{A}_{b_4^\dagger a_3^\dagger} &= \frac{\beta}{2}\Big[(\cos(\theta) - e^{i\phi}\sin(\theta))(i\sin(\theta) - ie^{i\phi}\cos(\theta))\Big].
\end{aligned} \tag{7}$$

Computing these terms and making the substitutions $\beta^2 = 1 - \eta^2$, $\eta^2 \to \alpha e^{-\tau^2/\sigma^2}$ and $\phi \to 2\pi c\tau/\lambda = 2\pi\tau\nu$ (where ν

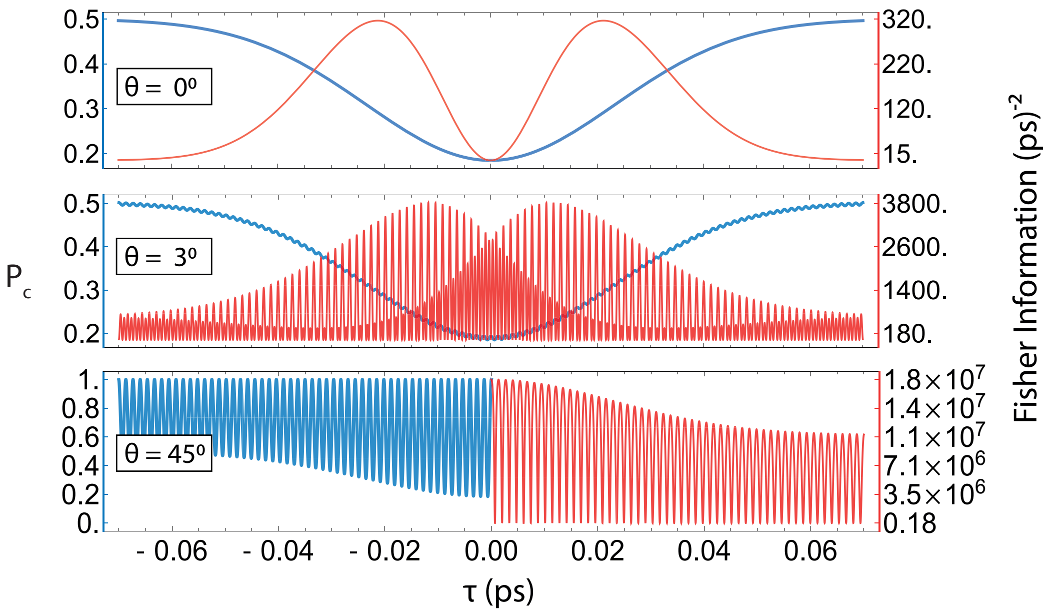

fig. S1. Predicted Fisher information for a HOM with added phase-dependent fringes. The visibility of the fringes can be tuned to increase the obtainable precision albeit at the expense of introducing potential instabilities and reducing the dynamic range. These plots correspond to $\alpha = 0.63$, $\sigma = 0.033$ ps, and $\nu = 371$ THz (no loss is modelled, so $\gamma = 0$). The curves in the lowermost panel have been alternately cutaway either side of the origin (for clarity). All curves are symmetric around $\tau = 0$.

is the frequency of the light), we have

$$\begin{aligned} P_c &= |\mathcal{A}_{a_3^\dagger a_4^\dagger} + \mathcal{A}_{a_4^\dagger a_3^\dagger}|^2 + |\mathcal{A}_{b_3^\dagger a_4^\dagger}|^2 + |\mathcal{A}_{b_4^\dagger a_3^\dagger}|^2 \\ &= \frac{1}{2}\left(\sin^2(2\theta)\left(\alpha e^{-\frac{\tau^2}{\sigma^2}}+1\right)\cos^2(2\pi\nu\tau) - \alpha e^{-\frac{\tau^2}{\sigma^2}} + 1\right). \end{aligned} \tag{8}$$

Note the $\cos^2$ function, indicative of N=2 N00N interferometry, which is easily switched on in our setup – this could provide higher information and a way to remove ambiguities whilst not sacrificing the dynamic range of the HOM setup by rotating the polarisation of the input photon pair before the PBS thereby switching between the two schemes. It should be noted that in this case the same level of stabilisation is required as classical interferometry. When $\theta \to 0$, we recover the usual HOM dip (see Figure S1). The full information is:

$$F = -\frac{4\left(\pi\nu\sigma^2\sin^2(2\theta)\left(\alpha + e^{\frac{\tau^2}{\sigma^2}}\right)\sin(4\pi\nu\tau) + \alpha\tau\sin^2(2\theta)\cos^2(2\pi\nu\tau) - \alpha\tau\right)^2}{\sigma^4\left(\alpha + e^{\frac{\tau^2}{\sigma^2}}\right)\left(\sin^2(2\theta)\cos^2(2\pi\nu\tau) - 1\right)\left(\sin^2(2\theta)\left(\alpha + e^{\frac{\tau^2}{\sigma^2}}\right)\cos^2(2\pi\nu\tau) - \alpha + e^{\frac{\tau^2}{\sigma^2}}\right)}. \tag{9}$$

In the case of $\theta \to 0$, this expression reduces to the lossless version of the Fisher information used in the main paper. In the case of $\theta \to 45°$, it simplifies to

$$F = \frac{8\left(\alpha\tau\sin(2\pi\nu\tau) - 2\pi\nu\sigma^2\left(\alpha + e^{\frac{\tau^2}{\sigma^2}}\right)\cos(2\pi\nu\tau)\right)^2}{\sigma^4\left(\alpha + e^{\frac{\tau^2}{\sigma^2}}\right)\left(\alpha(\cos(4\pi\nu\tau) - 1) + e^{\frac{\tau^2}{\sigma^2}}(\cos(4\pi\nu\tau) + 3)\right)}. \tag{10}$$

Setting $\alpha = 0.63$, $\sigma = 0.033$ ps, $\nu = 371$ THz, we find that the peak Fisher information for $\theta = 45°$ is approximately 24000 times higher that for $\theta = 0°$. This equates to a potential 155 fold improvement in precision.

List of fitting parameters and results

The table below shows the collection of parameters used to analyse the data shown in all figures along with the final results.

table S1. Summary of all measurements and parameters used in the fitting procedure. The final row corresponds to an average of the absolute value of each column. The factor 1×10^9 is denoted bn for billions. $n = 1$ or 1.5 is the refractive index of the sample material (air or glass respectively). The single datapoint relating to the glass wedges is highlighted with horizontal lines. Average and best case values are shown in bold face.

		$\delta\tau$ (attosecond)				$c\delta\tau/n$ (nanometre)				fitted parameters			
Figure	n	set	measured	accuracy	precision	set	measured	accuracy	precision	N	σ (ps)	γ	α
2	1.0	-	scan	-	-	-	scan	-	-	3.72e+5	0.07	0.88	0.53
2	1.0	-	scan	-	-	-	scan	-	-	3.72e+5	0.07	0.87	0.78
2	1.0	-	scan	-	-	-	scan	-	-	3.72e+5	0.06	0.87	0.92
3 and 4	1.0	-33.33	-28.97	-4.36	14.00	-10.00	-8.69	-1.31	4.20	434 bn	0.03	0.87	0.63
4	1.0	-66.67	-57.20	-9.47	46.18	-20.00	-17.16	-2.84	13.85	276 bn	0.05	0.87	0.65
4	1.0	-100.00	-96.27	-3.73	15.48	-30.00	-28.88	-1.12	4.64	287 bn	0.04	0.86	0.69
4	1.0	-6.67	-6.16	**-0.51**	13.41	-2.00	-1.85	-0.15	4.02	190 bn	0.03	0.87	0.63
4	1.0	-50.00	-60.84	10.85	30.29	-15.00	-18.25	3.25	9.09	63 bn	0.03	0.89	0.73
4 (wedges)	1.5	-56.67	-69.36	12.69	**4.72**	-11.33	-13.87	-2.18	0.94	414 bn	0.03	0.88	0.75
4	1.0	-16.67	-17.47	0.80	6.19	-5.00	-5.24	0.24	1.86	361 bn	0.03	0.87	0.65
4	1.0	-6.67	-16.68	10.02	7.41	-2.00	-5.01	3.01	2.22	148 bn	0.03	0.87	0.63
4	1.0	-1.67	-9.43	7.77	7.49	-0.50	-2.83	2.33	2.25	321 bn	0.02	0.87	0.69
4	1.0	-1.67	-6.36	4.69	25.13	-0.50	-1.91	1.41	7.54	502 bn	0.05	0.89	0.68
average	-	-	-	**6.48**	**17.03**	-	-	**1.78**	**5.06**	-	-	-	-
